# Atrial fibrillation and survival in colorectal cancer

**DOI:** 10.1186/1477-7819-2-40

**Published:** 2004-11-29

**Authors:** Stewart R Walsh, Kelly M Gladwish, Nicholas J Ward, Timothy A Justin, Neil J Keeling

**Affiliations:** 1 Department of Colorectal Surgery, West Suffolk Hospital NHS Trust, Hardwick Lane, Bury St Edmunds, Suffolk IP32 7TG, United Kingdom

## Abstract

**Background:**

Survival in colorectal cancer may correlate with the degree of systemic inflammatory response to the tumour. Atrial fibrillation may be regarded as an inflammatory complication. We aimed to determine if atrial fibrillation is a prognostic factor in colorectal cancer.

**Patients and methods:**

A prospective colorectal cancer patient database was cross-referenced with the hospital clinical-coding database to identify patients who had underwent colorectal cancer surgery and were in atrial fibrillation pre- or postoperatively.

**Results:**

A total of 175 patients underwent surgery for colorectal cancer over a two-year period. Of these, 13 patients had atrial fibrillation pre- or postoperatively. Atrial fibrillation correlated with worse two-year survival (p = 0.04; log-rank test). However, in a Cox regression analysis, atrial fibrillation was not significantly associated with survival.

**Conclusion:**

The presence or development of atrial fibrillation in patients undergoing surgery for colorectal cancer is associated with worse overall survival, however it was not found to be an independent factor in multivariate analysis.

## Background

In general, colorectal cancer patients are three times more likely to be in atrial fibrillation preoperatively than matched controls undergoing non-colorectal cancer surgery [1,2]. It may also occur postoperatively. Recent data suggest that atrial fibrillation may be an inflammatory complication, resulting from the initiation of an inflammatory response to surgery or infection [3-6]. Colorectal cancer patients may have elevated C-reactive protein (CRP) levels [7] indicating a systemic inflammatory response. Elevated CRP levels may be associated with a worse prognosis in colorectal cancer patients [8]. Postoperative dysrhythmias may [9] or may not [10] be associated with worse survival following surgery for lung cancer. We hypothesised that atrial fibrillation (AF) is an adverse prognostic indicator in patients undergoing surgery for colorectal cancer.

## Patients and methods

Patients who underwent a resection for colorectal cancer between 1^st ^January 2000 and 31^st ^December 2001 in a 600-bed district general hospital in the United Kingdom National Health Service were identified. The hospital serves a population of approximately 230,000. About 90 elective and emergency laparotomies are performed each year for colorectal cancer. Patients were identified from the prospectively maintained colorectal cancer database maintained by the colorectal surgical department. Patients with radiological, endoscopic or clinical examinations suspicious of colorectal cancer are referred to the weekly colorectal multi-disciplinary team (MDT) meeting. In the case of suspicious radiology, the referral to the MDT is made automatically by the radiology department. This avoids the possibility of the responsible clinical firm failing to refer a patient for consideration. Similarly, the pathology department automatically refers any patient in whom histology shows colorectal malignancy. In addition, patients who undergo surgery where a suspicion of colorectal cancer is raised are referred for consideration. The colorectal meeting is attended by the colorectal surgeons, radiologists, pathologists, palliative care physicians and nursing staff. Patients determined to have colorectal cancer by the MDT are entered into the database. The colorectal department periodically compares the database to clinical coding data for patients with colorectal cancer in order to ensure complete data capture. All patients are followed-up regularly by a team of colorectal nurse specialists in a dedicated clinic.

Age, sex, mode of presentation (emergency or elective), Dukes stage, postoperative anastomotic leakage and adjuvant therapy were recorded for all patients. The colorectal cancer database was cross-referenced with the hospital clinical-coding database to identify those patients who were in atrial fibrillation at any time before or after their surgery. Patients with colorectal cancer who did not undergo surgery or who only underwent palliative stoma formation were excluded. All patients were followed up for at least two years postoperatively. Overall survival and recurrence-free survival were recorded. Recurrence-free survival was defined as the time interval between operation and first diagnosis of local or distant recurrence. Patients with no recurrence were censored at the time of death from any cause other than cancer or at the time they were last seen by the colorectal team. Characteristics between those with and without AF were compared using the Student t-test and Fisher Exact test for continuous and categorical data respectively. Potential prognostic factors were compared by the log-rank test. Significant prognostic factors identified from the univariate analysis were entered into a multivariate Cox regression model of survival to test for independence. The 5% level was considered significant in the multivariate analysis. Statistical analysis was performed using Statsdirect^® ^version 2 (Statsdirect Ltd., UK).

## Results

One hundred and seventy-five patients (M:F = 111:64) who underwent bowel resection for colorectal cancer were identified from the database. Their median age was 74 years (interquartile range 66 to 80 years). Tumour site, Dukes stage and mode of operation (emergency or elective) are summarised in Table 1. Anastomotic leaks occurred in three patients while another three patients received preoperative radiotherapy. Median follow-up was 2.38 years. There were 60 deaths (42 cancer-specific deaths) during the follow-up period. The remaining 18 patients died from conditions such as pneumonia, pulmonary embolus or myocardial infarction. Cause of death was recorded for all patients in the database. Twenty-eight patients (16%) developed recurrence during postoperative surveillance. The remaining 14 patients who died were noted to have incurable disease at the time of surgery.

**Table 1:** Baseline characteristics of study group

	**No. of patients (%)**
Age	
≥72 years	98 (56%)
< 72 years	77 (44%)
Gender	
Male	111 (64%)
Female	64 (36%)
Site	
Colon	119 (68%)
Rectum	56 (32%)
Dukes' Stage	
A	26 (15%)
B	75 (43%)
C	57 (33%)
D	17 (9%)
Presentation	
Elective	147 (84%)
Emergency	28 (16%)

Cross-referencing with the clinical coding database identified thirteen patients with a history of atrial fibrillation. Five patients were in AF preoperatively. The remaining eight patients developed postoperative AF. A comparison of baseline characteristics between patients with and without AF is shown in table 2. The AF was paroxysmal in three patients, persistent but eventually resolved in five and permanent in the remaining five patients. There were seven deaths among the patients with atrial fibrillation: two from recurrent colorectal cancer, four from other causes (pneumonia in two patients, respiratory failure secondary to pulmonary fibrosis in one and left ventricular failure in one) with recurrent cancer present and one non-cancer related death (peri-operative myocardial infarction).

**Table 2:** Comparison of characteristics between patients with AF and those without.

**Characteristic**	**Sinus rhythm**	**Atrial fibrillation**	**p**
Male gender	102	9	0.77
Mean age	72	73	0.64
Rectal cancer	52	4	0.99
Elective surgery	139	8	0.05
Pre-operative radiotherapy	2	1	0.22
Anastomotic leak	3	0	0.79
Dukes Stage			
A	24	2	0.73*
B	69	6	
C	53	4	
D	16	1	

*Fisher-Freeman-Halton Exact Test

### Survival analysis

**Figure 1:**
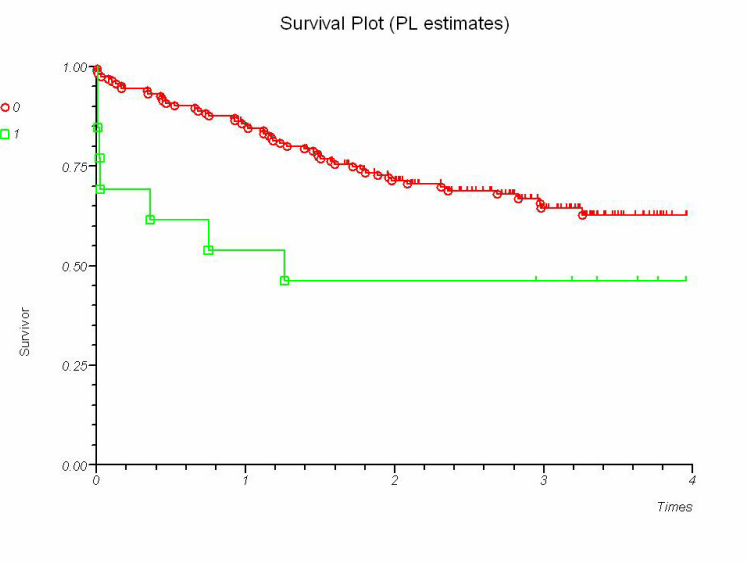
Kaplan-Meier survival curves for patients with a history of atrial fibrillation (1) versus those without (0). P = 0.04 (Log-rank test).

There was no correlation between overall survival and the following variables in the univariate survival analysis: gender, postoperative anastomotic leak, site of tumour (rectal versus colonic) or preoperative radiotherapy. Mode of surgery (emergency or elective), age (<72 years or ≥ 72 years) and Dukes' stage had a significant effect on survival. When patients with atrial fibrillation were compared to those without (Figure 1), atrial fibrillation correlated with worse overall survival. Dukes' stage, mode of surgery, age and atrial fibrillation were entered into a Cox regression model overall survival (Table 2). Mode of surgery, age and Dukes' stage retained significance but atrial fibrillation did not (Model chi-square 49.6; 7 degrees of freedom; p < 0.0001). There was no significant correlation between atrial fibrillation and recurrence-free survival (p = 0.74).

## Discussion

Tumours stimulate an inflammatory response when they invade or metastasise [11]. This inflammation may cause a rise in serum levels of inflammatory markers such as CRP. Serum CRP levels correlate with survival in colorectal cancer patients [8]. Atrial fibrillation may be precipitated or maintained by an inflammatory mechanism [3]. Thus, we hypothesised that the presence of AF, an inflammatory complication, would be associated with poorer survival in colorectal cancer as it is a manifestation of more advanced disease. Our data demonstrate a significant correlation between atrial fibrillation and survival following surgery for colorectal cancer in univariate analysis. Previous investigators have found that atrial arrhythmias were significantly more common in those patients with a history of cancer. In addition, CRP levels were higher in those patients with a history of cancer. However, there was no independent link between cancer and arrhythmias. This is consistent with inflammation being the causal link between cancer and arrhythmias [12].

In our cohort, atrial fibrillation was not an independent predictor of survival. Dukes' stage was the strongest predictor of survival. Previous data from colorectal cancer patients suggest the degree of inflammatory response may reflect the degree of disease progression [13]. The incidence of liver metastases, peritoneal tumour deposits, lymph node metastases and intravascular invasion are higher in patients with elevated CRP levels [14]. Thus, atrial fibrillation may be a manifestation of systemic inflammation due to more advanced disease and would not be independent of Dukes' stage. Cox regression analysis of our study cohort (Table 3) appears to show poor survival in Dukes B patients (Hazard ratio 9.13) compared to Dukes C patients (Hazard ratio 0.15). There was a trend for Dukes B patients to be older (mean age 73.79 years) than Dukes C patients (70.99 years; p = 0.1, student t-test, β = 0.67) and this may account for the reduced survival. There were no differences in the Dukes' stage distribution between patients with and without AF (Table 2). However, the small numbers in our series (only thirteen patients with AF) render it underpowered to detect a correlation between the presence of AF and colorectal cancer stage.

**Table 3:** Overall univariate and multivariate survival analysis

**Variable**	**Univariate p**	**Multivariate p**	**Hazard Ratio**	**95% Confidence interval of hazard ratio**
Age (< 72 years versus ≥ 72 years)	0.03	0.003	2.38	1.339 to 4.224
Pre-operative radiotherapy	0.26	-	-	-
Anastomotic leak	0.23	-	-	-
Gender (Male versus female)	0.33	-	-	-
Site of tumour (Rectal versus colonic)	0.87	-	-	-
Emergency surgery	<0.0001	0.004	2.35	1.312 to 4.205
Atrial fibrillation	0.04	0.06	2.21	0.979 to 4.985
Dukes' stage (A, B, C, D)	<0.0001	<0.0001	Dukes A 0.09	0.032 to 0.267
			Dukes B 9.13	4.343 to 19.206
			Dukes C 0.15	0.073 to 0.312
			Dukes D 1	Reference

There are several other limitations to our study. The quality of the clinical coding has not been assessed. Thus, the sensitivity and specificity of our clinical coding department in coding AF is unknown. Only 13 patients (7.4%) developed AF. Previous work suggests that 13% of elective colorectal patients develop postoperative AF [15]. Thus, it is possible that some patients with unrecorded AF have been incorporated into the control arm of our series. The study cohort has been followed for a relatively short time, only two years. However, most trials of follow-up following surgery for colorectal cancer report a mean time to relapse of approximately 24 months [16]. We were unable to demonstrate a significant relationship between atrial fibrillation and recurrence-free survival. This may be due to an insufficient sample size. However, atrial fibrillation does correlate with overall survival. The presence of atrial fibrillation may be a clinical marker of poor overall survival in patients undergoing surgery for colorectal cancer.

## Competing interest

The author(s) declare that they have no competing interests.

## Authors' contribution

**SRW **conceived the study, performed the statistical analysis and drafted the paper.

**KMG **and **NJW **performed the literature search and collected the data.

**TAJ **and **NJK **co-wrote the paper with **SRW**.

All authors approved the manuscript.

## Funding support

None declared
